# Supplementary material for: A UMLS-based spell checker for natural language processing in vaccine safety
Source: BMC Med Inform Decis Mak. 2007 Feb 12;7:3. doi: 10.1186/1472-6947-7-3 (PMC1805499; doi:10.1186/1472-6947-7-3)
Supplement: Additional file 13 — RAP application directory. Sets up directory for RAP (RDF application for PHP) source code [file 1472-6947-7-3-S13.gz › rap/doc/phpdoc/__filesource/fsource_dataset__datasetQuad.php.html]

xml version="1.0" encoding="iso-8859-1"?


File Source for Quad.php


# Source for file Quad.php

Documentation is available at Quad.php

```
1. <?PHP
2. // ----------------------------------------------------------------------------------
3. // Class: Quad
4. // ----------------------------------------------------------------------------------


8. /**
9. *
10. * A Triple in a RDF dataset, consisting of four Jena Nodes: graphName,
11. * subject, predicate, and object.
12. *
13. * <BR><BR>History:<UL>
14. * <LI>05-03-2005                : First version of this class.</LI>
15. *
16. * @version  V0.9
17. * @author Daniel Westphal (http://www.d-westphal.de)
18. *
19. * @package     dataset
20. * @access    public
21. ***/
22. class Quad
23. {

25. /**
26. * Name of the NamedGraphMem
27. *
28. * @var        Resource
29. * @access    private
30. */
31. var $graphName;

33. /**
34. * Statement
35. *
36. * @var        Statement
37. * @access    private
38. */
39. var $statement;

41. /**
42. * Constructor
43. * Creates a Quad from four Nodes.
44. *
45. * @param Resource
46. * @param Resource
47. * @param Resource
48. * @param Resource
49. * @access    public
50. */
51. function Quad($graphName,$subject,$predicate,$object)
52. {
53. if (!is_a($graphName, 'Resource'))
54. {
55. $errmsg = RDFAPI_ERROR .
56. '(class: Quad; method: new): Resource expected as graphName.';
57. trigger_error($errmsg, E_USER_ERROR);
58. }
59. $this->statement=new Statement($subject,$predicate,$object);
60. $this->graphName=$graphName;
61. }

63. /**
64. * Sets the graph name.
65. *
66. * @param Resource
67. * @access    public
68. */
69. function setGraphName($graphName)
70. {
71. $this->graphName=$graphName;
72. }

74. /**
75. * Return the graph name.
76. *
77. * @return Resource
78. * @access    public
79. */
80. function getGraphName()
81. {
82. return $this->graphName;
83. }

85. /**
86. * Return a human-readable (sort of) string "graphname { s p o . }"
87. * describing the quad.
88. * @return string
89. */
90. function toString()
91. {
92. return 'GraphName('.$this->graphName->getLabel().') '.$this->statement->toString();
93. }

95. /**
96. * Return the subject.
97. *
98. * @return Resource
99. * @access    public
100. */
101. function getSubject()
102. {
103. return $this->statement->getSubject();
104. }

106. /**
107. * Return the predicate.
108. *
109. * @return Resource
110. * @access    public
111. */
112. function getPredicate()
113. {
114. return $this->statement->getPredicate();
115. }

117. /**
118. * Return the object.
119. *
120. * @return Resource
121. * @access    public
122. */
123. function getObject()
124. {
125. return $this->statement->getObject();
126. }

128. /**
129. * Return the statement(subject,predicate,object).
130. *
131. * @return statement
132. * @access    public
133. */
134. function getStatement()
135. {
136. return $this->statement;
137. }

139. /**
140. * Checks, if two quads are equal.
141. *
142. * @param  Quad
143. * @return boolean
144. * @access    public
145. */
146. function equals($quad)
147. {
148. return ($this->graphName->equals($quad->getGraphName()) && $this->statement->equals($quad->getStatement()));
149. }
150. }
151. ?>
```

Documentation generated on Thu, 7 Jul 2005 13:42:38 +0200 by phpDocumentor 1.3.0RC3
